# Plasma cell-free DNA: a potential biomarker for early prediction of severe dengue

**DOI:** 10.1186/s12941-019-0309-x

**Published:** 2019-03-13

**Authors:** Nguyen Thi Ngoc Phuong, Dao Huy Manh, Shyam Prakash Dumre, Shusaku Mizukami, Lan Nguyen Weiss, Nguyen Van Thuong, Tran Thi Ngoc Ha, Le Hong Phuc, Tran Van An, Thuan Minh Tieu, Mohamed Gomaa Kamel, Mostafa Ebraheem Morra, Vu Thi Que Huong, Nguyen Tien Huy, Kenji Hirayama

**Affiliations:** 1 Department of Immunogenetics, Institute of Tropical Medicine (NEKKEN), Nagasaki University, Nagasaki, Japan; 2 Health Innovation Course, School of Tropical Medicine and Global Health, Nagasaki University, Nagasaki, Japan; 3 Global Leader Nurturing Program, Graduate School of Biomedical Sciences, Nagasaki University, Nagasaki, Japan; 4 Department of Immunology and Microbiology, Pasteur Institute, Ho Chi Minh City, Vietnam; 5 Nguyen Dinh Chieu Hospital, Ben Tre Province, Vietnam; 6 Online research Club (www.onlineresearchclub.org/), Nagasaki, Japan; 7 Faculty of Health Sciences, McMaster University, Hamilton, Canada; 8 Faculty of Medicine, Minia University, Minia, Egypt; 9 Faculty of Medicine, Alazhar University, Cairo, 11884 Egypt; 10 Evidence Based Medicine Research Group, Ton Duc Thang University, Ho Chi Minh City, Vietnam; 11 Faculty of Applied Sciences, Ton Duc Thang University, Ho Chi Minh City, 70000 Vietnam; 12 Department of Clinical Product Development, Institute of Tropical Medicine (NEKKEN), School of Tropical Medicine and Global Health, Nagasaki University, Nagasaki, 852-8523 Japan

**Keywords:** Severe dengue, Cell-free DNA, Prognostic biomarker, Dengue severity predictor

## Abstract

**Background:**

Considerable progress has been made in dengue management, however the lack of appropriate predictors of severity has led to huge number of unwanted admissions mostly decided on the grounds of warning signs. Apoptosis related mediators, among others, are known to correlate with severe dengue (SD) although no predictive validity is established. The objective of this study was to investigate the association of plasma cell-free DNA (cfDNA) with SD, and evaluate its prognostic value in SD prediction at acute phase.

**Methods:**

This was a hospital-based prospective cohort study conducted in Vietnam. All the recruited patients were required to be admitted to the hospital and were strictly monitored for various laboratory and clinical parameters (including progression to SD) until discharged. Plasma samples collected during acute phase (6–48 h before defervescence) were used to estimate the level of cfDNA.

**Results:**

Of the 61 dengue patients, SD patients (n = 8) developed shock syndrome in 4.8 days (95% CI 3.7–5.4) after the fever onset. Plasma cfDNA levels before the defervescence of SD patients were significantly higher than the non-SD group (*p* = 0.0493). From the receiver operating characteristic (ROC) curve analysis, a cut-off of > 36.9 ng/mL was able to predict SD with a good sensitivity (87.5%), specificity (54.7%), and area under the curve (AUC) (0.72, 95% CI 0.55–0.88; *p *= 0.0493).

**Conclusions:**

Taken together, these findings suggest that cfDNA could serve as a potential prognostic biomarker of SD. Studies with cfDNA kinetics and its combination with other biomarkers and clinical parameters would further improve the diagnostic ability for SD.

**Electronic supplementary material:**

The online version of this article (10.1186/s12941-019-0309-x) contains supplementary material, which is available to authorized users.

## Background

Dengue is a mosquito-borne viral disease of the tropics/subtropics caused by any of the four dengue virus serotypes (DENV-1 through -4) which is responsible for at least two million severe cases among the 96 million apparent infections every year globally [1–3]. Dengue clinical spectrum ranges from mild to severe dengue (SD). SD is described by the presence of severe plasma leakage, severe bleeding and organ impairment [2]. Mechanisms of dengue pathogenesis and severity are not clear, although several host (e.g. primary vs secondary immune response) and viral factors are considered accountable for progression to SD [3, 4].

No specific treatment is currently available for dengue and the recently licensed vaccine has limited efficacy [5]. Moreover, its geographical expansion has resulted in increased frequency and magnitude of epidemics and the escalating number of SD patients led to a huge economic burden worldwide [3]. Although the use of ‘warning signs’ has contributed significantly in clinical management, it is difficult to accurately recognize SD patients in early phase of disease using these warnings signs [6, 7]. Apparently, the use of warning signs as proxy indicators for admission has added extra burden to the hospitals, and more importantly, some dengue patients without warning signs may also progress to SD—a serious drawback of this system [2, 3]. Therefore, from the patient management point of view, early prediction of dengue severity could be a game changer in reducing hospital burden and mortality while enhancing the quality of care to the severe patients [8]. Unfortunately, there is no reliable routine prognostic test available yet [9]. There have been growing efforts made on the discovery of predictors based on severity biomarkers alone or in combination with dengue clinical signs [8–11], however these are either not validated clinically or the evidence is not adequate for clinical application [12–14].

Therefore, the quest/validation of biomarkers in dengue is well grounded. In the SD biomarker pipeline, circulating cell-free DNA (cfDNA) can be considered one of the potential candidates based on the evidences previously reported in other health conditions as highlighted herein. cfDNA is a double-stranded DNA (dsDNA) (mitochondrial or nuclear) fragment released in extracellular fluids from various cells [15, 16]. Apoptosis is believed to be the major source of cfDNA in plasma [17], although the exact mechanism of its generation is still enigmatic. Whatever the source of cfDNA, it could be a new avenue in dengue predictor studies. First, because cfDNA has been extensively studied in various cancer conditions [18, 19] and implemented as a potential marker [19–21]. Despite its application in cancer conditions as a biomarker, its utility is not adequately explored in viral diseases. Second, the association between apoptosis and dengue severity has been reported [22, 23], and in a preliminary study, our group indicated that the cfDNA level increased in severe patients, however its SD predictive potential was not validated in the early stage of the disease [24]. Therefore, it is important to identify the potential diagnostic role of cfDNA in early recognition of SD among dengue patients. In this study, we aimed to investigate the association of plasma cfDNA with SD, and evaluate whether cfDNA could be a predictive biomarker for SD at early acute phase of illness.

## Methods

### Ethics statement

This study was approved by the Institutional Review Boards of Pasteur Institute in Ho Chi Minh City (PIHCM), Vietnam (No. 602/QD-Pas 27/12/10), and Institute of Tropical Medicine, Nagasaki University, Nagasaki, Japan (No. 11063072) and conducted according to Helsinki Declaration with a written informed consent being obtained from each study participant and/or parent/primary caregiver.

### Study design and enrollment

This study was carried out using samples from dengue patients enrolled in a hospital-based prospective study at Nguyen Dinh Chieu Hospital, Ben Tre province, Vietnam from July 2011 to May 2013. Five years old or older patients admitted with a suspected acute dengue infection, presenting with acute onset of fever (≥ 38 °C within the last 72 h) and with no severe symptoms before hospital admission were included. Patients with known evidence or history of chronic diseases, cardiovascular disease, hypertension, respiratory disease, hepatitis, kidney impairment, gastric or duodenal ulcer, diabetes, osteoporosis, glaucoma, immunodeficiency disease, significant anemia (hemoglobin < 8 g/L), and immunosuppressive drugs use within the last two weeks of enrollment were excluded.

### Patient admission and diagnosis

All the recruited patients were required to be admitted in hospital for close monitoring despite no severe signs appearing at the time of admission. Non-structural (NS)-1 antigen test (Bio-Rad Laboratories Inc., Marnes-la-Coquette, France) positive patients were further confirmed by reverse transcription (RT) PCR and in-house IgM antibody capture enzyme linked immuno-sorbent assay (MAC-ELISA) or anti-dengue IgM/IgG ELISA as described previously (Additional file 1: Table S1) [24–27]. Primary and secondary dengue infections were determined using IgM/IgG ratios in acute and convalescent sera by capture ELISAs (Pasteur Institute, Vietnam). Secondary infection was defined when the IgM/IgG ratio was < 1.8 or had a positive IgG result in acute phase with subsequent ≥ 4- fold rise in convalescent sera [2, 24, 26]. Likewise, DENV IgM positive case was considered to be primary infection when the ratio of IgM/IgG was ≥ 1.8 or had a negative IgG result in acute phase [2, 28]. NS1 positive patients (except 2 NS1 negative but RT-PCR positive patients) were recruited to ensure active (current) DENV infection (Additional file 1: Table S1). Also, anti-DENV IgG detection (Pasteur Institute) remained helpful to rule out past infection [24, 26, 29]. Therefore, we considered all the included patients had active DENV infection. With thorough clinical examinations and DENV specific assays (antigen, antibody and viral RNA) in a dengue endemic setting, it was less likely to have infections other than DENV.

### Patient monitoring for disease progression and shock

All the admitted patients were strictly monitored everyday by experienced physician(s) for disease progression (clinical course) until discharge. Patients received standard treatment according to the guidelines of Ministry of Health, Vietnam. All the clinical data were duly recorded, which include but not limited to blood collection time, clinical manifestations (vital signs, vomiting, hemorrhagic tendencies (such as mucosal, gastrointestinal, menstruation, nosebleed, etc.), liver enlargement, and progression to severe syndromes, e.g. shock), treatment history, and laboratory parameters (such as hematocrit level, platelet count, leukocyte count, etc.).

**Fig. 1:**
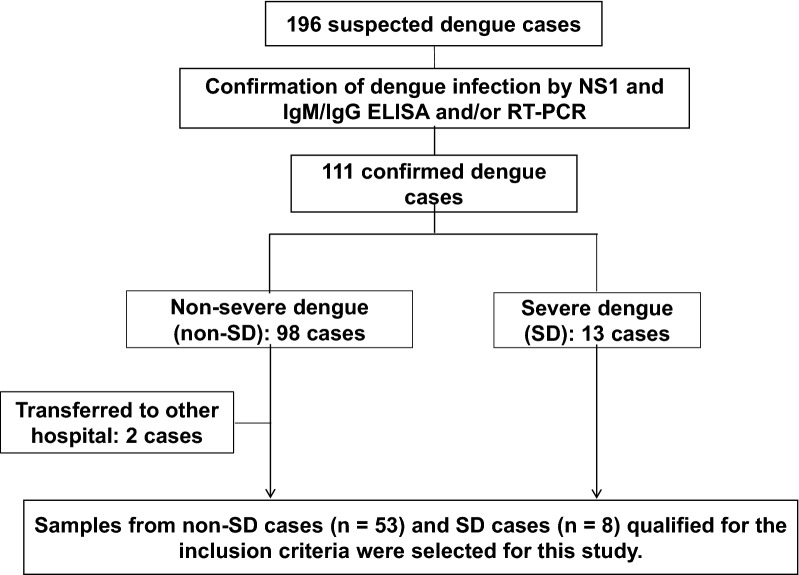
Flow-diagram of the case selection and processing. NS1, Non-structural protein 1; ELISA, Enzyme linked immuno-sorbent assay; PCR, Polymerase chain reaction

All the eligible patients admitted and monitored were classified according to the classification criteria of the World Health Organization (WHO)-2009 for severity grading (Fig. 1). Patients who developed severe manifestations like shock syndrome were considered as SD according to the WHO-2009 criteria while those who did not develop any severe forms were classified as non-SD (dengue without and with warning signs) [2]. Clinical outcome (e.g. shock) was then linked back to patient data.

Acute plasma samples (collected 6–48 h before defervescence) from eligible dengue patients were selected for this study and stored at − 80 °C. Additionally, control blood samples were taken from nine healthy Vietnamese donors (from the same Kinh ethnic group to eliminate potential ethnic and demographic bias) without current or recent history of fever or any other disease symptoms. Plasma samples from healthy volunteers were tested for DENV NS1 antigen, RNA and IgM antibody to rule out DENV infection as described above. These healthy plasma samples were exclusively used in the preparation of standard curve required for quantitative measurement of cfDNA in patient’s plasma samples as described elsewhere [24].

### Plasma cfDNA measurements

The cfDNA levels of acute-phase plasma samples were measured by Quant-iT™ PicoGreen^®^ dsDNA Reagent and Kits (Invitrogen, USA) with some modifications [30, 31]. With PicoGreen, dsDNA can be quantitated with very minimal interference (< 10%) by single-stranded DNA (ssDNA) or RNA in the sample [32]. Briefly, 3 μL of patient’s plasma was added into each micro-well containing 100 μL of TE buffer (10 mM Tris–HCl, 1 mM EDTA, pH 7.5) followed by addition of 100 μL of PicoGreen working solution. Reaction mixture was dark incubated for 5 min and fluorescence was measured (at 485 nm excitation, 535 nm emission wavelengths) using a fluorescence microplate reader (Perkin Elmer Wallac 1420). Standard curve (Additional file 1: Fig. S1) was created with known concentrations of Lambda DNA prepared in TE, 3 μL of which was added to well containing 97 μL TE and 3 μL healthy plasma. Healthy plasma (3 μL) in TE buffer (100 μL) was used as background. To resemble the plasma sample physiology, pooled plasma from nine healthy donors (with negligible DNA concentration) was used in TE buffer to prepare the standard curve. Each assay was performed in duplicate. Perfect linearity of the standard curve was observed in a range of 6.9–443.4 ng/mL. Concentrations of unknown plasma samples were determined by using the linear equation.

### Data analysis

Patient’s demographic, clinical and laboratory data were entered into spread sheet (master file) and underwent data cleaning/verification. Data were analyzed by GraphPad Prism software version 6.05. The cfDNA levels of each severity group were presented as median and interquartile range (IQR). Differences between two groups were analyzed by using Mann–Whitney *U* test. Cell counts and cfDNA concentration data were also subjected to Spearman’s correlation test. A *p* value < 0.05 was considered as statistically significant for all analyses. Receiver Operating Characteristic (ROC) curve was made and the area under ROC curve (AUC) was analysed to determine the discriminatory performance of cfDNA in predicting SD.

## Results

### Demographic and clinical profiles

The age of the dengue patients in our cohort ranged from 6 to 44 years (children ≤ 15 years, 65.6%), and the vast majority (78.7%) had secondary infection (Table 1). No significant difference was observed between SD and non-SD patients for most demographic (age, sex) and clinical (abdominal pain, persistent vomiting, mucosal bleeding, etc.) features, and laboratory findings (DENV serotypes, platelet counts).

**Table 1:** Clinical features and plasma cfDNA levels in patients with severe and non-severe dengue

Characteristics	Non-SD (n = 53)	SD (n = 8)	p-value*
Demographic features			
Age—median year (IQR)	12.0 (10.0–18.0)	12.5 (10.0–17.3)	0.9456
Male (%)	22 (41.5)	3 (37.5)	1
Clinical symptoms, physical signs and intervention			
Abdominal pain (%)	30 (56.6)	6 (75.0)	0.4525
Persistent vomiting (%)	4 (7.5)	2 (25.0)	0.1731
Mucosal bleeding (%)	7 (13.2)	3 (37.5)	0.1154
Liver enlargement > 2 cm (%)	1 (1.89)	1 (12.5)	0.247
Narrow pulse pressure	0	8 (100)	< 0.0001
Shock manifestation	0	8 (100)	< 0.0001
Infusion (Ringer’s lactate)	37 (69.8)	8 (100)	0.0097
Laboratory test			
Secondary infection (%)	41 (77.36)	7 (87.5)	1
PLT (× 10^3^/μL)—median (IQR)^a^	123.0 (92.0–165.5)	101.0 (48.3–147.3)	0.2784
Dengue serotype			
DENV-1 (%)	9 (17.0)	1 (12.5)	1
DENV-2 (%)	14 (26.4)	3 (37.5)	0.674
DENV-3 (%)	3 (5.7)	2 (25.0)	0.1239
DENV-4 (%)	15 (28.3)	1 (12.5)	0.6681
Unknown (%)	19 (35.8)	2 (25.0)	0.7028
Day of sampling from fever onset—median day (IQR)	3.0 (3.0–4.0)	3.0 (3.0–4.0)	0.7368
Time of sampling before defervescence—median hour (IQR)	20.0 (14.0–28.0)	15.5 (11.3–23.8)	0.2931
Plasma cfDNA levels, median (IQR), ng/mL	35.4 (24.4–51.6)	61.4 (38.3–110.5)	0.0493

SD, severe dengue; non-SD, non-severe dengue; IQR, interquartile range; PLT, platelet count; DENV, dengue virus; cfDNA, cell-free DNA

* Difference between categorical variables (sex, infection type, clinical signs, etc.) was determined by χ2 test or Fishers-exact test as appropriate, while the difference between continuous variables (age, platelet counts, sampling duration and cfDNA levels) was calculated by Mann–Whitney *U* test (between two groups) as appropriate. A p-value < 0.05 was considered significant

^a^Samples collected from 6 to 48 h before defervescence. PLT count for four patients was missing

### Dengue disease progression and clinical outcomes

Of the eligible dengue patients (n = 61) included in the present study, eight patients developed severe manifestations (shock syndrome) while the rest 53 remained as non-SD (Fig. 1) during the in-patient follow up. Detailed clinical information of each patient has been provided in supplementary materials as Additional file 2. Among the SD patients, defervescence was observed after 4.3 days (95% CI: 3.6–5.0; range: 3–5 days) of fever, and all eight patients developed shock between 3 and 6 days (mean [95% CI]: 4.8 days [3.7–5.4]) after the fever onset. The average time interval between defervescence and shock was 17.2 h (95% CI: 9.2–25.2; range: 8–26 h). All shock patients but one had secondary infection, and the shock patient with primary infection had DENV-1 and -3 co-infection.

### Plasma cfDNA levels remained significantly higher in SD patients during acute phase of illness

**Fig. 2:**
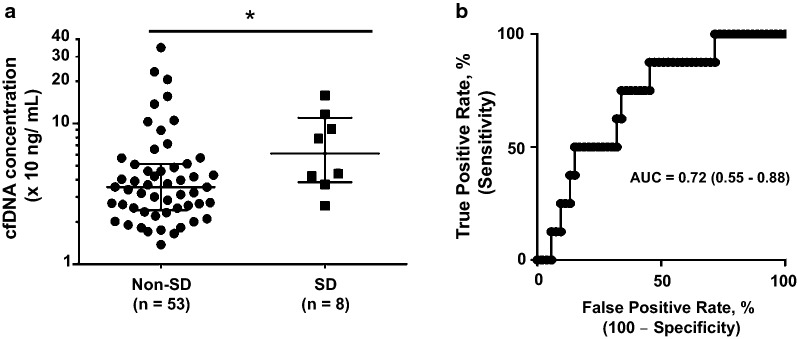
Acute phase plasma cfDNA levels associated with dengue severity. **a** Plasma cfDNA levels in patients with non-severe dengue (n = 53) and severe dengue (n = 8). The error bars represent the median and (^*^) indicates *p* < 0.05 by Mann–Whitney *U* test for continuous variables. cfDNA concentration is expressed on a log scale (Y-axis). **b** ROC curve of plasma cfDNA levels as a predictor of SD. Plasma cfDNA had an AUC of 0.72 (95% CI: 0.55–0.88; *p *= 0.0493). cfDNA, Cell-free DNA; ROC, Receiver operating characteristics; AUC, Area under the ROC curve

The acute plasma cfDNA levels were significantly higher (*p *= 0.0493) in the SD group [median (IQR): 61.4 ng/mL (38.3–110.5)] compared to the non-SD group [35.4 ng/mL (24.4–51.6)] (Table 1 and Fig. 2a). Plasma cfDNA had an AUC of 0.72 (95% CI: 0.55–0.88; *p *= 0.0493) in predicting SD (Fig. 2b). With a cut-off value > 36.85 ng/mL, cfDNA demonstrated sensitivity and specificity of 87.5% (95% CI: 47.4%–99.7%) and 54.7% (40.4%–68.4%) respectively, in predicting SD among the total dengue patients.

We did not find significant difference in the overall cfDNA level between primary and secondary DENV infection, and between the SD and non-SD secondary infections (Additional file 1: Fig. S2). This suggests that cfDNA does not vary between primary and secondary dengue infections.

**Fig. 3:**
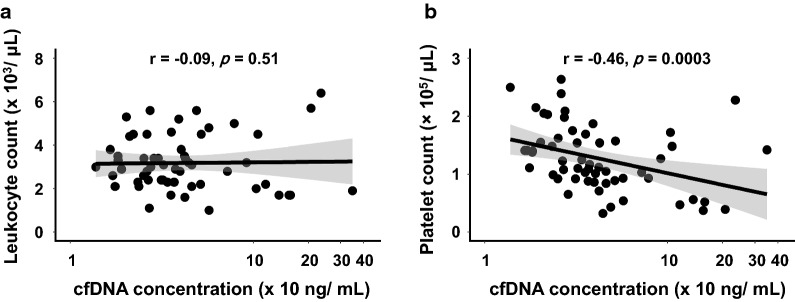
Correlation between plasma cfDNA level and leukocyte or platelet count in dengue patients. Leukocyte count (**a**) or platelet count (**b**) was plotted against the plasma cfDNA concentration (ng/mL) to determine their correlation by Spearman’s method. Correlation coefficient (r) of 1 or − 1 indicates a perfect correlation between two variables, while r = 0 indicates no correlation. In the scatter plot graph, data is presented as correlation line (bold straight line) and 95% confidence interval (CI) (shaded area). cfDNA concentration is expressed on a log scale (X-axis). Statistically significant correlation was considered, when *p* < 0.05. Leukocyte and platelet count data were missing for 4 cases, and hence the correlation was performed using data from 57 cases

On further analysis, we also observed significant correlation between cfDNA concentration and platelet count (r = − 0.46, *p* = 0.0003) but not the leukocyte count (r = − 0.09, *p* = 0.51) (Fig. 3).

## Discussion

Here, we report the potential of plasma cfDNA as an early predictor of SD along with clinical and laboratory findings in our cohorts. Identification of reliable biomarkers/predictors is crucial in dengue because neither the sole dependency on warning signs described in WHO guidelines [2, 33], nor other proposed algorithms are sufficient to predict SD during the early stage of illness [12–14].

We found significantly elevated levels of cfDNA (6–48 h before defervescence) in dengue patients who later progressed to SD compared to those who did not. More precisely, in predicting SD, the acute cfDNA alone exhibited a good sensitivity and specificity with an AUC > 0.7 (Fig. 2b) which is considered as acceptable predictive performance [34]. Still, warning signs are widely used in recognizing patients at risk of developing SD despite their subjective nature (some signs) and late appearance in the disease course [2]. This halts early SD detection and timely management, and is also criticized for over-estimation of SD [33]. In that sense, cfDNA is a simple tool, likely makes the SD prediction more practical and explicit as anticipated in our study previously [24]. Moreover, the use of cfDNA in combination with other early signs probably further enhance the SD prediction. Several candidates have been studied to explore severity predictors in dengue such as vascular endothelial growth factor (VEGF), tryptase and chymase [35, 36], transforming growth factor-beta (TGF-b), and VEGF receptor-2 [9], cytokines (IL-10, IFN-γ) [8] and plasma IgE levels [11]. For instance, we recently reported the ratio of DENV specific IgE and total IgE (S/T ratio) as a potential candidate predictor (sensitivity/specificity, 75%/68%) [11]. In our preliminary analysis, another candidate biomarker TGF-b-induced protein (TGFBIp) has also shown some promising results (data not shown). Therefore, the combination of cfDNA with other potential candidate biomarkers [8, 9, 11, 35, 36] or clinical signs is worthwhile when applied to recently proposed prediction models [12–14, 37]. Yet, none of these this combination was investigated in our cohort partly due to the small number of samples in the severe group, as we enrolled patients at early state which resulted into fewer patients in the SD group.

Regardless of the difference in cfDNA levels between SD and non-SD groups, the underlying mechanism and its role in the SD pathogenesis are not clear. Apoptosis being the major source of cfDNA in bloodstream [17] and its presence in various tissues from fatal dengue patients suggests involvement of apoptosis in SD pathogenesis [22]. The apoptotic microvascular endothelial cells probably play roles in vascular permeability—a hallmark of SD [22]. The level of peripheral mononuclear cell apoptosis around defervescence also well correlated with SD in children [23]. In addition, high mobility group box 1 (HMGB1) [38], TGF-beta [39], TNF-alpha, nitric oxide and NS1 [4, 40] related to apoptosis were reported at increased levels in SD patients’ samples, further supporting the role of apoptosis in SD and cfDNA as its proxy indicator. Undoubtedly, the knowledge on the source of this cfDNA would aid in the further understanding of pathogenesis too.

We also found a significant correlation between decreasing platelet count and increasing cfDNA concentration in dengue patients. Probably, it is associated with binding and subsequent platelet activation by DENV as reported previously [2, 41, 42]. Platelet activation is believed to release mitochondria [43], leading to elevated mitochondrial DNA in plasma [44], which in turn perhaps contributed to high cfDNA levels in dengue patients with decreasing platelet counts. Mitochondrial DNA released from platelet is also a potent inflammatory trigger which causes cytokines release and systematic inflammation [43]. This pro-inflammatory response might also play a role in dengue clinical outcomes.

Besides, the failure to remove cfDNA from the bloodstream was also explained by severe multi-organ dysfunctions (liver and kidneys), one of the dengue severe forms [24], however the SD patients in this study did not present these manifestations. Since the half-life of cfDNA in the circulation is short [21], the levels of cfDNA in dengue patients might be fluctuated over time. Though all the samples in this study were selected early before defervescence/shock, the sampling time frame might be broad. Therefore, additional studies to investigate the kinetics of cfDNA in different time points of the disease course will be informative to select the best sampling time for measuring cfDNA to precisely predict SD. As a limitation, we did not measure the cfDNA in other severe conditions/infections, thus it requires cautious interpretation among dengue patients co-infected with other pathogens.

We are aware that the cfDNA assay may not be promptly applicable to the clinical settings in its current form and it certainly requires additional prospective studies in larger cohorts before moving to clinical application. This cfDNA assay is very simple, rapid, inexpensive and efficient (3 μL plasma volume). We have further simplified this assay by eliminating the need of enzymatic digestion used previously [24]. Requirement of a fluorescence microplate reader could limit its wide application for now. However, we believe that cfDNA is one of the simple assays and with the current technological advancement, it is very likely to be developed as simpler formats (even field-friendly devices) in the future for use in clinical settings in combination with other predictors (if not adequate alone).

## Conclusions

To our knowledge, the use of cfDNA for predicting dengue severity has not been reported earlier, although there are reports on the prognostic value of cfDNA in other conditions [21, 45, 46]. In conclusion, our findings demonstrated that plasma cfDNA levels could be used as a potential predictor of SD in acute phase of illness. Since the removal of cfDNA from the bloodstream is rapid [21], further prospective studies with a larger sample size should be done to investigate cfDNA kinetics and combined with other early clinical parameters of dengue patients would improve its diagnostic ability for SD.

## Additional files

**Additional file 1: Table S1.** Diagnostic assay results of the eligible dengue patients (n = 61). **Fig. S1.** Standard curve plot of Lambda DNA. **Fig. S2.** cfDNA levels between primary and secondary dengue infection.

**Additional file 2.** The demographic, laboratory and clinical details of all eligible study participants including their diagnosis and clinical course information.

## Abbreviations

AUC: Area under the ROC
cfDNA: Cell-free DNA
CI: Confidence interval
DENV: Dengue virus
HMGB: High mobility group box
IQR: Interquartile range
NS: Non-structural
PIHCM: Pasteur Institute in Ho Chi Minh City
ROC: Receiver operating characteristic
RT: Reverse transcription
SD: Severe dengue
WHO: World Health Organization
