# Supplementary material for: Plasma cell-free DNA: a potential biomarker for early prediction of severe dengue
Source: Ann Clin Microbiol Antimicrob. 2019 Mar 13;18:10. doi: 10.1186/s12941-019-0309-x (PMC6419393; doi:10.1186/s12941-019-0309-x)
Supplement: Supplementary file 1 — Additional file 1: Table S1. Diagnostic assay results of the eligible dengue patients (n = 61). Fig. S1. Standard curve plot of Lambda DNA. Fig. S2. cfDNA levels between primary and secondary dengue infection. [file 12941_2019_309_MOESM1_ESM.pdf]

Ann Clin Microbiol Antimicrob

Additional file 1

Additional table (Table S1) and figures (Fig. S1 and S2)

Plasma cell-free DNA: a potential biomarker for early prediction of severe dengue

Nguyen Thi Ngoc Phuong[1,2,†], Dao Huy Manh[1,3,†], Shyam Prakash Dumre[1†], Shusaku Mizukami[1,4], Lan Nguyen Weiss[5], Nguyen Van Thuong[5], Tran Thi Ngoc Ha[1], Le Hong Phuc[6], Tran Van An[6], Thuan Minh Tieu[7,8], Mohamed Gomaa Kamel[7,9], Mostafa Ebraheem Morra[7,10], Vu Thi Que Huong[5], Nguyen Tien Huy[4,7,*], Kenji Hirayama[1,*]

[1]Department of Immunogenetics, Institute of Tropical Medicine (NEKKEN), Nagasaki University, Nagasaki, Japan; [2]Health Innovation course, School of Tropical Medicine and Global Health, Nagasaki University, Nagasaki, Japan; [3]Global Leader Nurturing Program, Graduate School of Biomedical Sciences, Nagasaki University, Nagasaki, Japan; [4]Department of Clinical Product Development, Institute of Tropical Medicine (NEKKEN), Nagasaki University, Nagasaki, Japan; [5]Department of Immunology and Microbiology, Pasteur Institute, Ho Chi Minh City, Vietnam; [6]Nguyen Dinh Chieu Hospital, Ben Tre Province, Vietnam; [7]Online research Club (www.onlineresearchclub.org/); [8]Faculty of Health Sciences, McMaster University, Hamilton, Canada; [9]Faculty of Medicine, Minia University, Minia, Egypt; and [10]Faculty of Medicine, Alazhar University, Cairo, 11884, Egypt.

[†]These authors contributed equally to this work.

***Correspondence to:** Kenji Hirayama (E-mail: hiraken@nagasaki-u.ac.jp) or Nguyen Tien Huy (E-mail: tienhuy@nagasaki-u.ac.jp), Department of Immunogenetics or Department of Clinical Product Development, Institute of Tropical Medicine, Nagasaki University, 1-12-4 Sakamoto, Nagasaki 852-8523, Japan.

Additional table S1

Diagnostic assay results of the eligible dengue patients (n = 61)

**Assay performed**	**Assay results, n (%)**		
	**Positive**	**Negative***	**Total**
NS1	59 (96.7)	2 (3.3)	61 (100)
RT-PCR	39 (63.9)	22 (36.1)	61 (100)
ELISA	44 (72.1)	17 (27.9)	61 (100)

*Two cases negative by NS1 were confirmed positive by RT-PCR. A total of 27 patients were positive by both RT-PCR and ELISA, and the remaining patients had positive results by either ELISA or RT-PCR. All the healthy control samples (n = 9) were tested negative by all of these three assays.

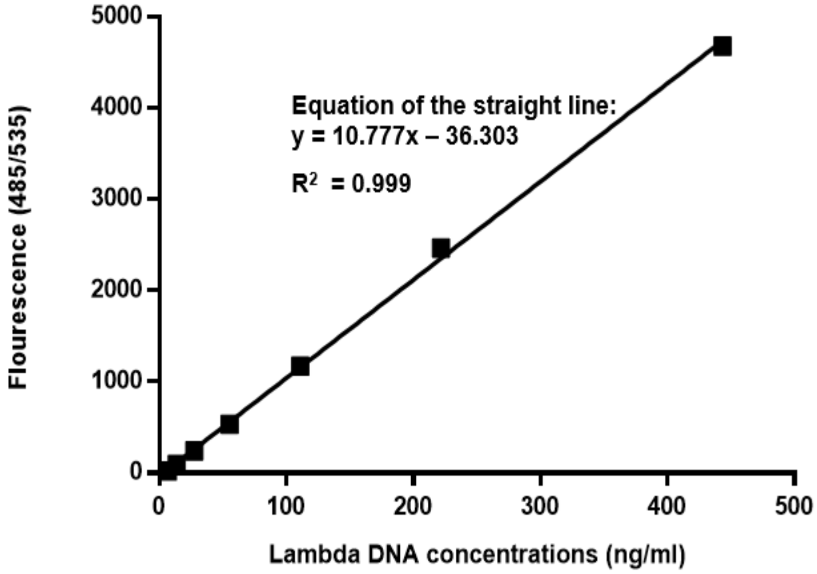

Additional fig. S1

Standard curve plot of Lambda DNA. To create the standard curve, known concentrations of Lambda DNA (X-axis) were used and after 5 minutes of dark incubation with PicoGreen working solution, the fluorescence intensity (Y-axis) measured at 485 nm excitation and 535 nm emission using fluorescence microplate reader. The linearity of standard curve was found in the range of 6.9 to 443.4 ng/mL. The resulting equation: $y = 10.777x - 36.303$, $R^2 = 0.999$ was used to estimate the DNA concentration of samples.

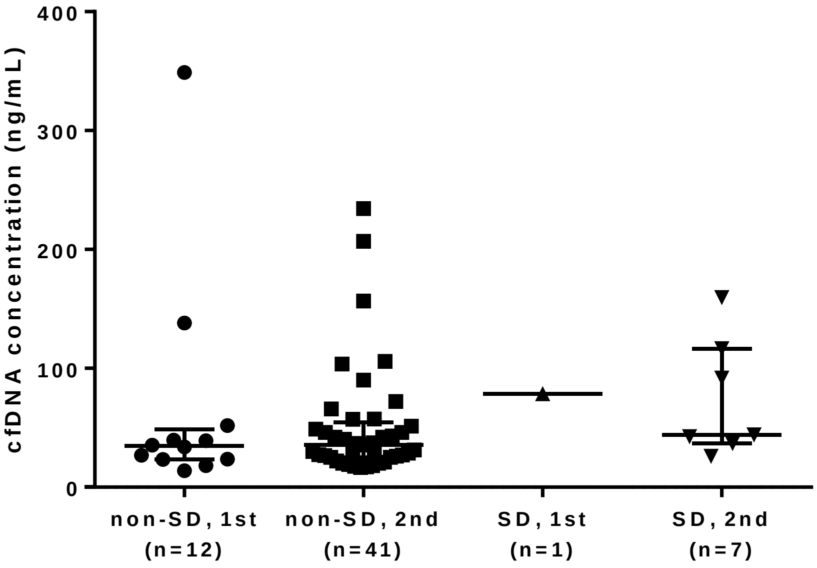

Additional fig. S2

cfDNA levels between primary and secondary dengue infection. Comparison was not done for primary infection with severe dengue due to sample number (n = 1)

1st = primary dengue infection; 2nd = secondary dengue infection

Additional file 2

Supplementary data set on demographic, laboratory and clinical details of all eligible patients (n = 61) including their diagnosis and clinical course information.

This data has been provided as a separate Excel sheet (**Additional file 2.xlsx**).
